# Low Serum Levels of Myeloid Progenitor Inhibitory Factor-1 Predict Good Response to Methotrexate in Rheumatoid Arthritis

**DOI:** 10.1155/2013/460469

**Published:** 2013-12-24

**Authors:** Varun Dhir, Amit Sandhu, Nidhi Gupta, Veena Dhawan, Shefali Sharma, Aman Sharma

**Affiliations:** ^1^Department of Internal Medicine (Rheumatology Unit), Post Graduate Institute of Medical Education and Research, Chandigarh 160012, India; ^2^Department of Experimental Medicine and Biotechnology, Post Graduate Institute of Medical Education and Research, Chandigarh 160012, India

## Abstract

*Background*. Although the benchmark in the treatment of rheumatoid arthritis remains methotrexate, only 70% of patients respond. Thus, there is a need for predictive biomarkers. This study planned to evaluate serum levels of myeloid progenitor inhibitory factor-1 (MPIF-1) and monocyte chemoattractant protein 2 (MCP-2)—as biomarkers. *Methods*. Patients with rheumatoid arthritis (RA) having high disease activity (DAS28-3v ≥ 5.1) were treated with oral methotrexate (MTX) for 12 weeks. Disease activity was measured by DAS28-3v (Modified Disease Activity Score 3 variables). Serum samples were stored at baseline and 12 weeks. *Results*. This study included 46 patients (F : M = 35 : 11) having mean (±SD) age of 42.6 ± 11.3 yrs, disease duration of 4.7 ± 4.5 yrs, and DAS28-3v of 6.1 ± 0.8. Serum MPIF1 was elevated in patients compared to controls (1636.7 ± 1009.7, 441.2 ± 173.8 pg/mL, *P* < 0.001), but there was no difference in MCP2 levels (31.4 ± 11.9, 33.8 ± 24.0 pg/mL). Baseline MPIF-1 level was lower in good responders (ΔDAS28-3v ≥ 1.2, *N* = 9) compared to poor responders (ΔDAS28-3v < 0.6, *N* = 27) (1171.0 ± 670.8, 1816.7 ± 1154.1 pg/mL, *P* = 0.05). On ROC analysis, baseline MPIF1 performed reasonably to predict good response; that is, ΔDAS28-3v ≥ 1.2 (AUC 0.68, 95% CI 0.50–0.87). *Conclusions*. Lower baseline MPIF1 level predicted a good response to methotrexate at 12 weeks.

## 1. Introduction

Rheumatoid arthritis is an autoimmune chronic inflammatory disease predominantly affecting the joints [1]. There is a recent emphasis on a “treat to target” strategy, with regular monitoring of disease activity and quick adjustments of drugs [2]. Although joint assessment remains the gold standard for disease activity, biomarkers may come to play a complementary role [3]. In the treatment of RA, the benchmark drug remains methotrexate. However, it is effective in only 70% of patients, and an excellent response is seen in only 30% of patients. A common therapeutic strategy is to start with methotrexate, and patients who do not respond to methotrexate are subsequently shifted to other drugs or combinations (other DMARDs or biologicals). However, waiting for response to methotrexate may delay institution of other effective therapies in nonresponders and contribute to joint damage. Thus, there is a need to identify nonresponders to methotrexate upfront to institute other therapies [4] hence the need for activity and predictive biomarkers [5].

Chemokines are small proteins involved in chemoattraction of a variety of cells to site of inflammation, being important in the pathogenesis of RA and may serve as biomarkers [6]. MCP-2 (monocytes chemoattractant protein 2) or CCL8 (chemokine C-C motif Ligand 8) is a chemokine involved in chemoattraction of monocytes, T cells, and NK cells [7, 8]. MPIF-1 (myeloid progenitor inhibitory factor-1) or CCL23 (chemokine C-C Ligand 23) is involved in chemoattraction of resting T cells and monocytes [9, 10]. Both of these have been shown to be upregulated in a previous study in RA and are potential biomarkers [11]. We wanted to look at their value as biomarkers in a prospective cohort of rheumatoid arthritis patients treated with methotrexate.

## 2. Material and Methods

This study was conducted from July 2011 to March 2012 at a university hospital after approval from the Institutional Ethics Committee. Written informed consent was taken from all subjects.

### 2.1. Study Design

This was a prospective study over 12 weeks, which included rheumatoid arthritis patients taking part in another clinical trial which compared starting with 7.5 mg versus 15 mg per week of methotrexate (Trial Registry no. NCT01404429). In this study only those patients who started with 15 mg methotrexate were included after consent.

### 2.2. Subjects

Inclusion criteria were (a) fulfillment of the 1987 American College of Rheumatology (ACR) criteria for rheumatoid arthritis [12], (b) 18 to 65 years of age, (c) having high disease activity (DAS28-3v ≥ 5.1), (d) not taking methotrexate for ≥2 months, (e) permitted to be on corticosteroids if dose is stable for ≥1 week and dose of prednisolone equivalent was ≤10 mg/day, and (f) permitted to be on other disease modifying antirheumatic drugs (DMARDs) like sulfasalazine, leflunomide, and hydroxychloroquine, if dosages are stable for ≥2 weeks. Standard exclusion criteria for methotrexate were followed. Patients were excluded if they were/had (a) breast-feeding or pregnant (in women), (b) liver disease, (c) renal disease, (d) active infections or (e) hepatitis B or C positive. In addition, 16 age- and gender-matched controls were enrolled from attendants of patients/staff after consent.

### 2.3. Intervention

Patients were started on 15 mg per week dose of methotrexate, which was escalated by 2.5 mg biweekly (maximum 25 mg) till 12 weeks. In addition, folate supplementation (5 mg twice a week = 10 mg/week) was given. Patients were monitored 4 weekly with complete blood counts and liver function tests, and methotrexate was withheld/not increased in case of cytopenias or transaminitis.

### 2.4. Clinical Assessment

The modified Disease Activity Score using 3 variables (DAS28-3v) was calculated to measure the disease activity [13]. This was calculated using the formula: DAS28-3v = [0.56*√ (tender joints) + 0.28*√ (swollen joints) + 0.70* Ln (ESR)] *1.08 + 0.16, where joint count was done on 28 joints and ESR was measured using Westegren method (first hour). Change in disease activity (∆DAS28-3) was calculated as the difference in DAS28-3 at baseline (0 week) “minus” final (12 weeks). Patients were stratified into 3 categories of response based on change in disease activity (∆DAS28-3). The three categories were good, moderate, and poor responsers, defined as ∆DAS28-3 ≥ 1.2, 0.6–1.2, and <0.6, respectively. In addition, the functional status of the patients was measured using Indian health assessment questionnaire at baseline and 12 weeks [14].

### 2.5. Laboratory Tests

Blood sample was obtained at baseline and 12 weeks; serum was separated and stored at −80°C. In addition, serum of controls was also stored. ELISA for MPIF-1 and MCP-2 (RayBio) was done on the stored samples after completion of original study. Minimum detectable limits of MPIF-1 and MCP-2 were 7 pg/mL and 1.5 pg/mL, respectively.

### 2.6. Statistical Analysis

Student's *t* test was used to compare the chemokine levels between patients and controls. Pearson's correlation was used to look at the correlation between chemokine levels and disease activity. In addition, multivariable linear regression and receiver operator curve (ROC) analysis were done. Analysis was done using SPSS v15 and GraphPad Prism (version 5).

## 3. Results

This study included 46 patients (F : M = 35 : 11) with rheumatoid arthritis. Their mean age was 42.6 ± 11.3 yrs and duration of disease was 4.7 ± 4.5 years. Baseline disease activity (DAS28-3v) was 6.1 ± 0.8, HAQ score was 1.3 ± 0.7, and 30 (65%) were rheumatoid factor positive. At 12 weeks, the mean dose of methotrexate reached was 24.3 ± 2.0 mg/week and mean (±SD) change in DAS28-3v and HAQ was 0.5 ± 0.6 and 0.3 ± 0.5, respectively.

**Figure 1:**
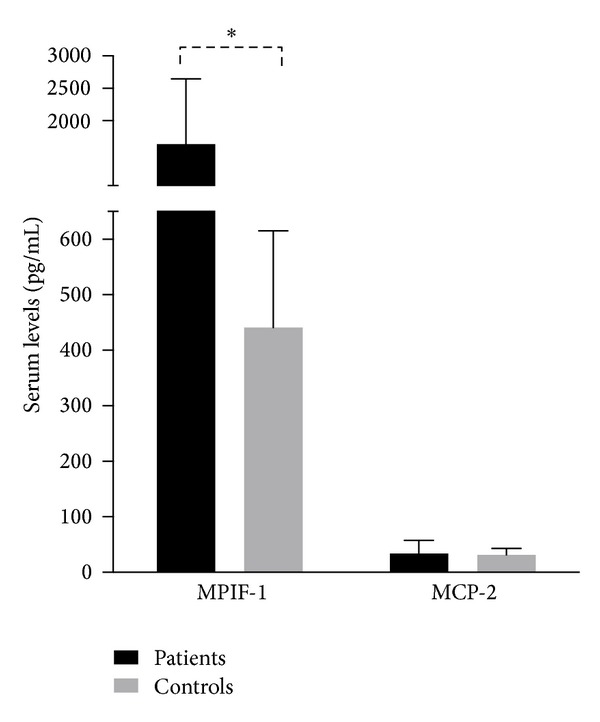
Baseline values of biomarkers (*P < 0.001).

**Figure 2:**
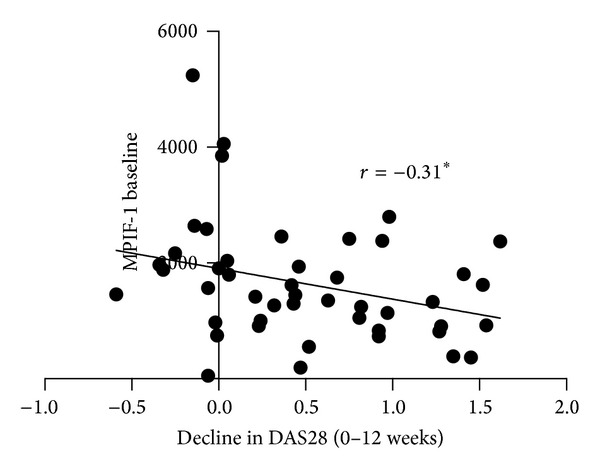
Correlation between baseline MPIF-1 and change in DAS28-3v (*P = 0.04).

Baseline level of MPIF-1 was elevated in patients compared to controls (1636.7 ± 1009.7, 441.2 ± 173.8, *P* < 0.001). However, there was no difference in the MCP-2 level (33.8 ± 24.0, 31.4 ± 11.9, *P* = ns) (Figure 1). On stratifying patients by response to MTX as per ∆DAS28-3v, among different baseline characteristics, only MPIF-1 level was significantly different across groups (Table 1). Also, baseline MPIF-1 had a significant though modest correlation with ∆DAS28-3v (*P* = 0.04) (Figure 2). On multivariate linear regression, only baseline MPIF-1 levels and disease duration were significant predictors of change in DAS28-3v (*r* ^2^ = 0.28). On ROC analysis, baseline MPIF-1 had area under curve of 0.68 (95% CI 0.50–0.87) and a level of <946 pg/mL was found to have the best predictive value with a sensitivity and specificity of 55.6 and 81% to predict good response to MTX.

Although baseline MPIF-1 was elevated and predicted response, it did not significantly change after 12 weeks (1557.4 ± 1155.4, *P* = ns). However, MCP-2 level increased at 12 weeks (55.0 ± 19.2, *P* < 0.001). There was a lack of correlation between change in DAS28 (∆DAS28-3v) and change in MPIF-1 levels (∆MPIF-1) (*r* = −0.24, *P* = 0.1) or MCP-2 levels (∆MCP-2) (*r* = −0.144, *P* = 0.35).

## 4. Discussion

This study found serum level of MPIF-1 to be raised in rheumatoid arthritis patients compared to controls. A lower level of MPIF-1 at baseline predicted better response to methotrexate over 12 weeks.

This study found MPIF-1 (myeloid progenitor inhibitory factor-1) levels to be raised 4-fold in serum of rheumatoid arthritis (RA) patients compared to controls. This is similar to a previous study that found a 1.3-fold higher level in plasma of patients [11]. Higher levels are not surprising, in view of the fact that MPIF-1 is involved in chemoattraction of resting T cells and monocytes [9, 10]. Also, its cognate receptor (CCR1) has been shown to be upregulated in synovial tissue of patients with rheumatoid arthritis [15].

Lower MPIF-1 levels at baseline were associated with better response to methotrexate at 12 weeks. Indeed, MPIF-1 was modestly accurate on ROC analysis (AUC = 0.68) in identifying patients with good response (∆DAS28 ≥ 1.2). Although it had modest predictive ability, as a comparison, a recent prediction model to predict methotrexate response in JIA, using SNPs in 4 genes and ESR, could only have an AUC of 0.65 [16]. This is the first study to look at MPIF-1 as a predictor for methotrexate response. In general, there is a paucity of biomarkers to predict response to methotrexate, and a systematic review found that predictive criteria were mainly clinical including male gender, low disease activity at baseline, DMARD naivety, negative rheumatoid factor, and being nonsmokers. However, none of these were found to have a high predictive value [17]. Indeed, these clinical parameters are probably markers of severe disease rather than of response to methotrexate [18]. Another study also reiterated that clinical characteristics were poor predictors of methotrexate response [19]. In contrast to our results of MPIF-1 as a predictive biomarker, we did not find it to be a useful biomarker for disease activity and found no change in levels on methotrexate treatment. A previous study also did not find any change in levels after 1 week of anti-TNF treatment, although they did find higher levels in active patients [11].

This study did not find any difference in MCP-2 levels between patients and controls, similar to a previous study [11]. However, MCP-2 has been found to be overexpressed in synovial biopsy specimens, [15] and its transcripts are upregulated on TLR2 stimulation of cultured synovial fibroblasts [20]. Thus, circulating MCP-2 levels may not reflect local synovial tissue levels. This study found an increase of MCP-2 levels posttreatment of rheumatoid arthritis (with decline in disease activity). A previous study, on the contrary, found higher levels in active patients versus quiescent patients. This difference can be partly explained by differences in design.

The strength of our study is the longitudinal design. Important drawbacks are the limited number of patients and the short followup of only 12 weeks. This short duration was chosen, as 12 weeks is fast emerging as a decision point as per both guidelines and major trials, when other drugs (including biologicals) are added in the face of inadequate response to MTX [21–23]. We preferred to use DAS28-3v score omitting the patient assessment on visual analog scale, as our patients poorly understand the concept of a visual analog scale.

To conclude, this study found that MPIF-1 may be a useful biomarker for predicting response to methotrexate in rheumatoid arthritis, with lower levels baseline predicting higher change in DAS28-3v (better response).

## Figures and Tables

**Table 1:** Baseline characteristics of the 3 categories of change in DAS28-3v.

Baseline characteristics ((mean ± SD), unless specified)	Change in DAS28-3v (0–12 weeks)		
	≥1.2 (*N* = 9)	0.6 to 1.2 (*N* = 10)	<0.6 (*N* = 27)
MPIF-1 (pg/mL)	1171.0 ± 670.8*	1570.0 ± 728.3	1816.7 ± 1154.1
MCP-2 (pg/mL)	29.5 ± 18.3	29.2 ± 22.2	36.7 ± 27.2
DAS28-3v	5.9 ± 0.9	6.3 ± 0.8	6.1 ± 0.7
ESR (mm first hour)	57.2 ± 31.2	77 ± 34.9	60.5 ± 31.3
Duration of disease (years)	3.8 ± 3.9	4.1 ± 3	6 ± 5.1
Age (years)	43.7 ± 10.4	42.3 ± 8.2	42.4 ± 12.8
RF positive *N* (%)	8 (89)	6 (60)	16 (59)
Female gender *N* (%)	5 (56)	9 (90)	21 (78)

*P = 0.05 compared to DAS28-3v < 0.6.
